# Advances in micro-/nanorobots for cancer diagnosis and treatment: propulsion mechanisms, early detection, and cancer therapy

**DOI:** 10.3389/fchem.2025.1537917

**Published:** 2025-02-06

**Authors:** Baiyang Fu, Dan Luo, Chao Li, Yiwen Feng, Wenlong Liang

**Affiliations:** ^1^ Department of Breast Surgery, The Second Affiliated Hospital of Harbin Medical University, Harbin, China; ^2^ College of Automotive and Mechanical Engineering, Harbin Cambridge University, Harbin, China; ^3^ Department of Rheumatology and Immunology, Daqing Oilfield General Hospital, Daqing, China; ^4^ Key Laboratory for Micro/Nano Technology and System of Liaoning Province, Dalian University of Technology, Dalian, China

**Keywords:** micro-/nanorobots, propulsion mechanisms, drug delivery, cancer diagnosis, targeted therapy

## Abstract

In recent years, medical micro-/nanorobots (MNRs) have emerged as a promising technology for diagnosing and treating malignant tumors. MNRs enable precise, targeted actions at the cellular level, addressing several limitations of conventional cancer diagnosis and treatment, such as insufficient early diagnosis, nonspecific drug delivery, and chemoresistance. This review provides an in-depth discussion of the propulsion mechanisms of MNRs, including chemical fuels, external fields (light, ultrasound, magnetism), biological propulsion, and hybrid methods, highlighting their respective advantages and limitations. Additionally, we discuss novel approaches for tumor diagnosis, precision surgery, and drug delivery, emphasizing their potential clinical applications. Despite significant advancements, challenges such as biocompatibility, propulsion efficiency, and clinical translation persist. This review examines the current state of MNR applications and outlines future directions for their development, with the aim of enhancing their diagnostic and therapeutic efficacy and facilitating their integration into clinical practice.

## 1 Introduction

Despite the remarkable advancements in the diagnosis and treatment of malignant tumors over the past decades, cancer remains the second leading cause of death in developed countries (Sung et al., 2021). There are still some limitations in the current diagnosis and treatment of cancer, including (Sung et al., 2021): The sensitivity of imaging or laboratory tests is insufficient, making it difficult to detect some early malignant tumors (Jelski and Mroczko, 2022; Vasireddi and Nguyen, 2021; Jelski and Mroczko, 2022) Traditional biopsy and surgery are difficult to operate at the single-cell level and cannot obtain tumor heterogeneity information (Kapoor-Narula and Lenka, 2022; Lou et al., 2022; Vasireddi and Nguyen, 2021) The non-specific delivery of anti-tumor drugs leads to the inability to target tumors and inevitable adverse reactions (Babiker et al., 2018; Di Nardo et al., 2022; Kapoor-Narula and Lenka, 2022) Malignant tumors can be insensitive or resistant to conventional chemotherapy, targeted therapy, and immunotherapy, potentially leading to disease progression (Lim and Ma, 2019; Richardson et al., 2023; Vesely et al., 2022). To overcome the above difficulties, medical micro-/nanorobots (MNRs) that can swim freely and operate in an orderly manner at the micrometer or nanometer scale have been designed by researchers.

Medical MNRs are defined as unconstrained micro- and nanostructures. MNRs contain drive structures that can convert various energy sources into driving forces and perform medical activities (Go et al., 2022; Li Y. et al., 2023; Soto et al., 2020). At present, based on the development and cross-integration of multiple disciplines such as micro/nano processing, biomaterials, and biomedicine, scientists have developed a variety of MNRs (Alizadeh et al., 2020; Yan et al., 2017; Song et al., 2021; Zhang W. et al., 2023). Medical MNR has basic features such as small size, wireless control, and payload capacity, which can achieve functions such as improving the sensitivity and specificity of tumor diagnosis, accurately targeting tumors, and operating at the single-cell level (Deng et al., 2020; Han et al., 2016; Li et al., 2017). However, it is still a huge challenge to apply MNRs in clinical tumor diagnosis and treatment.

To design smart MNRs for cancer patients, the following points still need to be considered. First, it is essential to take into account the physiological environment and the driving mode of MNR. Due to the extremely small size of MNR, its Brownian motion in the body environment is very strong, and it is extremely challenging to propel it in a low Reynolds number environment (Wu et al., 2022; Li J. et al., 2016). Therefore, a specific method is needed to propel and control the movement of MNR. Currently, the commonly used driving methods include non-toxic chemical fuels, external field sources, bioenergy, and hybrid driving modes (Cong et al., 2022; Li Z. et al., 2016; Ressnerova et al., 2021; Zhu et al., 2021). Secondly, the materials selected for MNRs should have the characteristics of low toxicity, good biocompatibility, degradability, and safe excretion, to ensure that the MNRs themselves will not affect the normal structure and function of the body (Kim et al., 2020). In addition, when designing micro-/nanorobots, researchers should consider the functions that the robots perform in the body and how they can continuously convert different forms such as magnetic energy, acoustic energy, and bioenergy into kinetic energy, and design the structures based on these requirements (Alapan et al., 2020). Finally, the size of the MNR and its ability to break through human barriers need to be taken into consideration. Nano drug carriers must overcome multiple barriers in the body, such as the vascular barrier, the blood-brain barrier, and the mucus-bicarbonate barrier on the surface of the gastric mucosa before they can reach the intended target site (Wang J. et al., 2022). This review mainly summarizes the latest progress in the driving modes and diagnostic and therapeutic applications of micro-/nanorobots in the diagnosis and treatment of malignant tumors, and discusses the current challenges and solutions in this field, providing ideas for the future development of new micro-/nanorobots that can be used in clinical practice.

## 2 Driving force type of micro-/nanorobot

### 2.1 Chemical fuel-driven micro-/nanorobot

**FIGURE 1:**
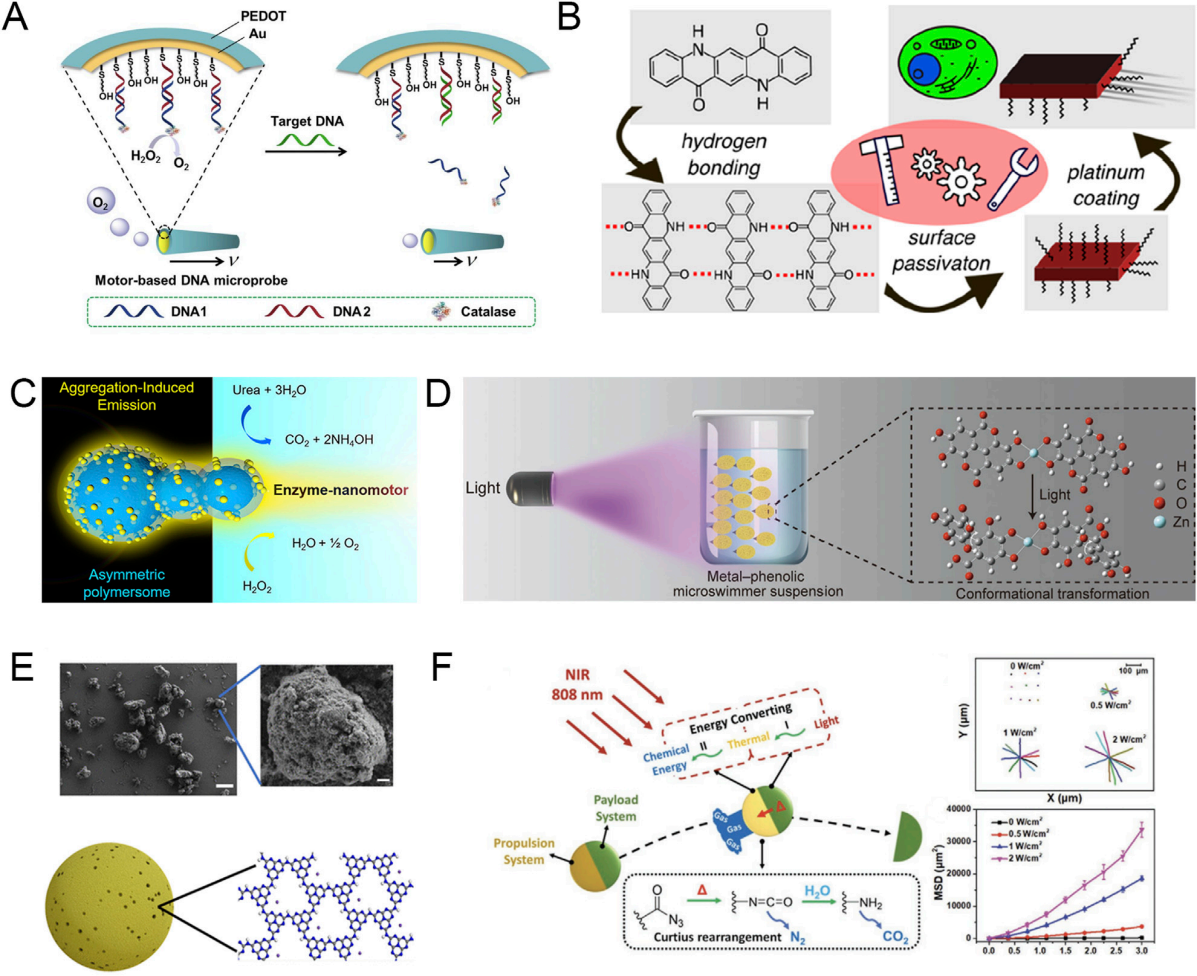
**(A)** Schematic diagram of the functions performed by the chemical fuel-driven motor microprobe. Modified and reprinted from ref (Xie et al., 2017). Reproduced with permission, Copyright 2016, Elsevier. **(B)** Schematic diagram of the microrobots exhibiting autonomous motion in the presence of hydrogen peroxide. Modified and reprinted from ref (Jancik-Prochazkova et al., 2023). Reproduced with permission, Copyright 2022, American Chemical Society. **(C)** Schematic diagram of a gourd-shaped polymer nanomotor with enzyme-powered motion, which generates motion through the enzyme-catalyzed decomposition of urea and hydrogen peroxide. Modified and reprinted from ref (Cao et al., 2021a). Reproduced with permission, Copyright 2021, The Authors. Published by American Chemical Society. **(D)** Schematic illustration of the light-induced motion of the metal–phenolic microswimmers. Modified and reprinted from ref (Lin et al., 2021). Reproduced with permission, Copyright 2021, Wiley-VCH. **(E)** SME images and schematic diagram of PHI (scale bars, 5 μm in A left and 400 nm in A right). Modified and reprinted from ref (Sridhar et al., 2022). Reproduced with permission, Copyright 2022, The Authors, some rights reserved; exclusive licensee American Association for the Advancement of Science. **(F)** Schematic diagram of NIR-driven nanorocket self-propulsion, propulsion trajectory and mean square displacement. Modified and reprinted from ref (Feng et al., 2023). Reproduced with permission, Copyright 2023, Wiley-VCH.

Chemical fuel propulsion refers to the reaction between a catalyst loaded on MNRs and the chemical “fuel” in the surrounding environment, which converts chemical energy into kinetic energy, enabling the efficient propulsion of the MNRs (Figure 1A) (Cai et al., 2023a; Cai et al., 2023b; Zhan et al., 2019).

As a chemical “fuel” in the environment, H₂O₂ has the characteristics of high reactivity and rapid bubble generation in the presence of catalysts, which makes H₂O₂ widely used in the early research on chemical fuel-driven micro-/nanorobots (Peng et al., 2023). For example, researchers have developed quinacridone and indigo-based microparticles and coated the microparticles with an asymmetric platinum layer with a thickness of 30 nm to prepare microrobots (Jancik-Prochazkova et al., 2023). The asymmetric coating on the robot can decompose H₂O₂ into water and oxygen, allowing the pigment-based Janus microrobot to move in the presence of H₂O₂. At the same time, the researchers found that increased H₂O₂ concentration and ultraviolet (UV) irradiation can increase the average movement speed of the microrobot, which can reach 2.8 ± 0.5 μm/s at the fastest. Compared with static particles, the chemical fuel-driven microrobot is more likely to accurately target, internalize, and accumulate in cells, thereby achieving its killing function (Figure 1B).

Although the increase in H₂O₂ concentration enhances the mobility of MNRs, high concentrations of H₂O₂ are potentially toxic to the human body, which limits the application scenarios of H₂O₂ in the body. Therefore, it is necessary to find chemical “fuels” that exist naturally in organisms or are harmless to organisms. Nowadays, researchers have discovered a variety of highly biocompatible chemical “fuels” such as water (Wavhale et al., 2021), urea (Choi et al., 2022), and collagen (Ramos-Docampo et al., 2019) that can be used for MNR propulsion. For example, Cao et al. developed a nanomotor that can use urea as a fuel for propulsion (Cao et al., 2021a). The study describes a supramolecular strategy for fabricating structural intrinsic fluorescence aggregation-induced emission (AIE) polymersome nanomotors with a gourd-like topology. Intrinsically asymmetric AIE polymersomes are decorated with thin polymer layers via a layer-by-layer supramolecular assembly approach, during which urease is encapsulated and embedded via strong electrostatic interactions. Urease can consume urea in its environment, thereby generating a concentration gradient and active directional flow on the particle surface, ultimately driving the nanomotor motion (Figure 1C).

Compared with normal cells, tumor cells need to take up a lot of glucose and are in a unique acidic environment (Wang Y. et al., 2022; Feng et al., 2024). These characteristics inspired researchers to design MNRs using the glucose and pH gradients naturally occurring in organisms. For example, Ji et al. designed the PDF@JAu@GOx nanomotor, whose propulsion mechanism is based on the glucose concentration gradient in the tumor microenvironment, which uses enzymatic reactions to decompose glucose to produce propulsion bubbles (Ji et al., 2024). This enhanced diffusion movement effectively increases the tumor cell uptake of the nanomotor, and the nanomotor can then exert its efficient delivery capacity.

Chemical “fuel” drive is a driving mode that can move autonomously and perform functions without external force. Although chemical “fuel” driven MNRs have broad prospects in the diagnosis and treatment of malignant tumors, there are still some problems to be solved. Since high concentrations of H₂O₂ are toxic, the efficiency of enzymatic reactions needs to be improved when designing MNRs that use H₂O₂ as fuel to achieve effective propulsion under low H₂O₂ concentration and dosage conditions. In view of the potential toxicity of H_2_O_2_ as a “fuel”, in our opinion, the development of more biocompatible “fuels” (such as water, urea, collagen, etc.) has wider application prospects. In addition, in the diagnosis and treatment of malignant tumors, if the glucose and pH concentration gradients in the tumor microenvironment can be cleverly used to design MNRs, it can not only enhance the effect in tumor treatment, but also reduce side effects on healthy tissues through high selectivity and controllability, thereby improving the safety and efficacy of treatment.

### 2.2 External field-driven micro-/nanorobot

#### 2.2.1 Light

Light-field driven optically controlled MNR refers to the light acting on the photosensitive units or structures of the MNR to produce specific reactions, such as temperature gradient, interfacial tension gradient, bubbles, and other reactions, which can realize the propulsion of the MNR (Hong et al., 2010; Villa et al., 2019; Cao et al., 2021b). Compared with other propulsion modes, light propulsion has attracted more attention due to its non-invasive, long-range, fuel-free, and relatively clean characteristics. In addition, the intensity, direction, time, and polarization of light are easy to adjust, which gives light-controlled MNRs higher controllability and freedom (Wang et al., 2018). In particular, light-activated nanomotors driven by photothermal conversion elements can ablate tumor cells while delivering drugs, which expands the application scenarios of light-driven MNRs (Huang et al., 2022).

Lin et al. reported a metal (Zn^2+^)-ellagic acid particle microswimmer that can autonomously sense and swim toward an external UV light source (Lin et al., 2021). Under a microscope, it was observed that exposure of the particle microswimmer to UV light caused it to move toward the illumination focus. When the light was enhanced, the speed of the microswimmer could reach 102 μm/s, and the directional movement of the microswimmer could be repeatedly activated and deactivated by turning the light source on and off. To further understand the phototaxis of the microswimmer, the researchers used five solutions of different polarities to conduct experiments and found that the speed of the microswimmer was inversely proportional to the polarity of the solution, which suggests that the positive phototaxis of the microswimmer may be driven by the conformational transition of ellagic acid at the molecular level (Figure 1D). Sridhar et al. developed a polyheptazine imide (PHI) microswimmer that can be driven by visible light (Sridhar et al., 2022). Under the irradiation of 415 nm blue light with an intensity of 0.42W/cm^2^, the PHI microswimmer can be proposed at a speed of 7.2 ± 1.1 μm/s in the DMEM culture medium. At the same time, considering the actual medical application scenarios, the researchers proposed that a light source can be provided in the deep area of the body through a catheter to guide the PHI microswimmer to be deployed at the target location to overcome the challenge of limited penetration of visible light (Figure 1E).

Although there are many studies on ultraviolet and visible light-driven MNRs, the limited penetration depth of these two types of light cannot be effectively solved (Sridhar et al., 2022). Therefore, near infrared (NIR) that can penetrate several centimeters of tissue may be more suitable for medical scenarios. For example, Feng et al. demonstrated a nanorocket (NR) with an asymmetric geometry (Feng et al., 2023). The NR can be remotely controlled by 808 nm NIR, which triggers photothermal conversion and Curtius rearrangement within the particle, thereby strongly releasing nitrogen to achieve ultrafast propulsion of nearly 300 μm/s. This powerful propulsion allows the NR to break through the physiological barriers in the tumor microenvironment and reach the target lesion directly (Figure 1F).

Light field-driven MNRs have high flexibility, improving the ability of light sources to penetrate deep tissues and reducing the damage of the light source itself to normal tissues are issues that must be studied before this driving mode can be applied clinically.

#### 2.2.2 Ultrasound

Ultrasound is an external field that is widely used in clinical diagnosis and treatment (Bachu et al., 2021). Compared with light fields, ultrasound has stronger tissue penetration ability, excellent biosafety, and lower cost. As a common external field, ultrasound can propagate in solids, liquids, and gases, and can also penetrate biological tissues to safely and effectively drive MNRs *in vivo* (Wang et al., 2019).

Wang et al. demonstrated an asymmetric needle-shaped MNR (Wang et al., 2020). The prepared MNR is a sound field-driven leukocyte membrane-coated gallium nanoswimmer (LMGNS). The speed of LMGNS is related to the ultrasonic frequency of the ultrasonic field. In the frequency range of 415–425 GHz, the average speed of LMGNS can reach more than 100 μm/s. In addition, the frequency of ultrasound can also adjust the movement direction of LMGNS. When the ultrasonic frequency drops from 420 GHz to 410 GHz, two LMGNS moving in opposite directions and moving away from each other can rotate 180° in 1.3 s and move in the opposite direction. At the same time, due to the characteristics of LMGNS such as strong absorption ability in the near-infrared region and excellent drug loading capacity, it exhibits a strong anti-cancer effect.

**FIGURE 2:**
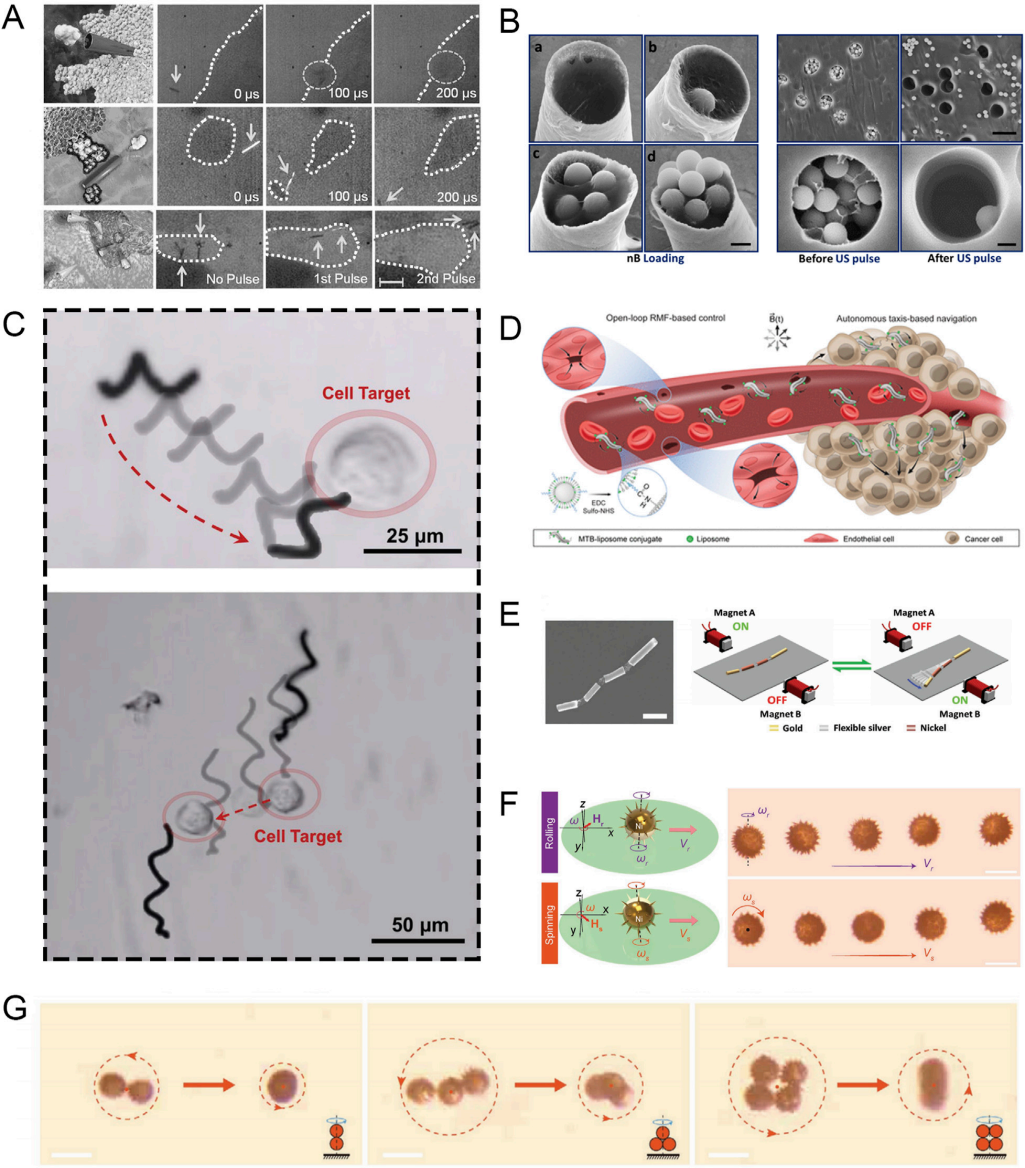
**(A)** Microbullets penetrate, deform, and cut tissue in response to US pulse signals (scale bars, 100 µm in a, 40 µm in b, and 80 µm in c). Modified and reprinted from ref (Kagan et al., 2012). Reproduced with permission, Copyright 2012, WILEY-VCH. **(B)** SEM micrographs showing Nb loading density and emission characteristics (scale bars, 1 μm in A, 10 μm in B top, and 1 μm in B bottom). Modified and reprinted from ref (Soto et al., 2016). Reproduced with permission, Copyright 2015, American Chemical Society. **(C)** MoSBOT single-cell manipulation under magnetic actuation. Modified and reprinted from ref (de la Asunción-Nadal et al., 2022). Reproduced with permission, Copyright 2022, The Authors. Small published by Wiley-VCH. **(D)** Schematic diagram of MTB crossing the vascular endothelial barrier and colonizing the tumor. Modified and reprinted from ref (Gwisai et al., 2022). Reproduced with permission, Copyright 2022, The Authors, some rights reserved; exclusive licensee American Association for the Advancement of Science. **(E)** SEM image of the multi-chain artificial nanofish and schematic diagram of magnetic propulsion of the nanofish by a planar oscillating magnetic field (scale bars, 800 nm). Modified and reprinted from ref (Li T. et al., 2016). Reproduced with permission, Copyright 2016, WILEY-VCH. **(F)** Motion characteristics of a single microswimmer (scale bars, 30 μm). Modified and reprinted from ref (Sun et al., 2020). Reproduced with permission, Copyright 2020, WILEY-VCH. **(G)** Collective motion characteristics of magnetic microswimmers (scale bars, 50 μm)**.** Modified and reprinted from ref (Sun et al., 2020). Reproduced with permission, Copyright 2020, WILEY-VCH.

In recent years, micromachines that use the rapid expansion and evaporation of perfluorocarbon droplets for high-speed propulsion have received considerable attention. Ultrasound in an external field can trigger the electrostatically bound perfluorocarbon droplets inside the machine, allowing the micromachine to obtain significant mechanical thrust, with an average speed of up to 6.3 m/s. Kagan et al. reported this ultrasound-triggered micromachine propulsion strategy, and the microbullet can achieve penetration, deformation, and cutting of sheep kidney tissue (Figure 2A) (Kagan et al., 2012). Soto et al. demonstrated an acoustically controlled microcannon (Mc) that can effectively load and launch nanobullets (Nb) (Soto et al., 2016). The electrochemically synthesized hollow Mc was loaded with a gel matrix containing Nb and perfluorocarbon emulsion. Ultrasound can trigger the rapid evaporation of the perfluorocarbon emulsion, resulting in the rapid ejection of Nb, similar to the barrel firing a bullet, with an average velocity of 1.05 ± 0.26 m/s (Figure 2B).

Although ultrasound-propelled MNRs have powerful penetration and propulsion capabilities, there are still some limitations to be addressed. For example, the geometry of the MNR needs to be designed appropriately to achieve efficient propulsion (Yu et al., 2024). It is necessary to select an appropriate ultrasonic power range to achieve high-speed and efficient propulsion without damaging tissue.

#### 2.2.3 Magnetic fields

Magnetically controlled MNR means that under the control of an external magnetic field, the MNR can convert the magnetic energy it receives into mechanical energy to achieve propulsion (Gong et al., 2022; Li T. et al., 2023). Compared with other external fields, magnetic fields have the characteristics of remote operation, high flexibility, and high biocompatibility, which makes external magnetic fields a common driving method for manipulating MNRs (Chen et al., 2021; Dogan et al., 2022; Park et al., 2021). To manufacture magnetically driven MNRs, magnetic materials are usually added to the surface or inside of the MNRs to achieve unique magnetically driven motion (Ussia et al., 2023; Wang et al., 2023). Although traditional static magnetic fields can allow magnetic micro-nano materials to move, the speed and direction of movement are poorly controlled (Liu et al., 2020). Dynamic magnetic fields (such as rotating magnetic fields and oscillating magnetic fields) can achieve remote and precise control, which has advantages that static magnetic fields cannot match (Yang and Zhang, 2020; Ji et al., 2021; Yu et al., 2019).

The researchers designed a MoS_2_-based microrobot (MoSBOT) that can achieve effective movement under a rotating magnetic field. To produce MoSBOT, the researchers adopted a bio-template method, using spirulina as a scaffold, and after creating a suitable Fe_3_O_4_ magnetic chassis, they used a hydrothermal reaction to evenly distribute the MoS_2_ nanosheets throughout the spiral structure. This allows MoSBOT to achieve propeller-like movement at a rotating magnetic field frequency of 3–6 Hz, and to achieve sufficient speed and controllability. In addition, MoS_2_ is a well-known photothermal material. The same MoSBOT can exhibit excellent ablation capabilities when triggered by NIR and can achieve similar effects to the most advanced anti-cancer drugs (Figure 2C) (de la Asunción-Nadal et al., 2022). Swiss researchers have found that compared with directional magnetic fields, uniform rotating magnetic fields (RMF) can enhance the ability of magnetotactic bacteria (MTB) to cross the vascular endothelial barrier, thereby enhancing the tumor colonization ability of anticancer therapeutic bacteria (Gwisai et al., 2022). By constructing a tissue barrier model to evaluate, it was found that the mechanism by which MTB translocation is enhanced in the presence of RMF is that the translational motion driven by the torque of the cell interface leads to increased surface exploration, making it easier to pass through any gaps between cells (Figure 2D).

Li et al. demonstrated a nanofish that uses a vibrating magnetic field for propulsion. This new nanoswimmer consists of gold segments as the head and tail, and two nickel segments as the body, and all segments are connected by three flexible porous silver hinges. Under the action of a vibrating magnetic field, the wave motion of the nanofish from head to tail is activated in sequence to generate a backward propagating wave, which can produce a traveling wave motion of 30 μm/s (Figure 2E) (Li T. et al., 2016).

In biomedical applications, the ability to deploy numerous microrobots simultaneously is often essential, highlighting the importance of studying the collective behavior of MNRs (Yu Y. et al., 2022). Sun et al. reported a sea urchin-like microswimmer based on sunflower pollen (Sun et al., 2020). When different input rotating magnetic fields are applied, the nickel coating gives each microswimmer two different motion modes (rolling and rotation). These two modes can be used for drug delivery and cell drilling, respectively. In addition, multiple individual microswimmers can form a kayak-mode polymer. The principle is that as the distance between microswimmers decreases, the fluid flow field coupling effect becomes greater. In addition, in the rotation mode, the polymer can stand up and transform from a 2D state to a 3D state as the input frequency increases (Figures 2F, G).

In summary, external magnetic field drive has good operability, and the variety and functions of magnetically controlled MNRs make magnetically controlled drive the most common drive mode in biomedical applications. However, current research on magnetically controlled actuation is mostly limited to the operation of a single MNR. In order to enable MNRs to play a more powerful clinical diagnosis and treatment role, the collective motion behavior of MNRs still needs further exploration.

### 2.3 Biologically driven micro-/nanorobot

Although researchers have developed a wide variety of chemical fuels and external field-driven micro-/nanorobots, the biocompatibility and degradability of micro-/nanorobots in the body are still unavoidable issues. Therefore, researchers use bacteria and cells in nature and in organisms to develop bioenergy-driven micro-/nanorobots. Directed movement can be achieved by utilizing the tropism of bacteria and cells, and preset functions can be realized (Chu et al., 2022; Suh et al., 2019).

**FIGURE 3:**
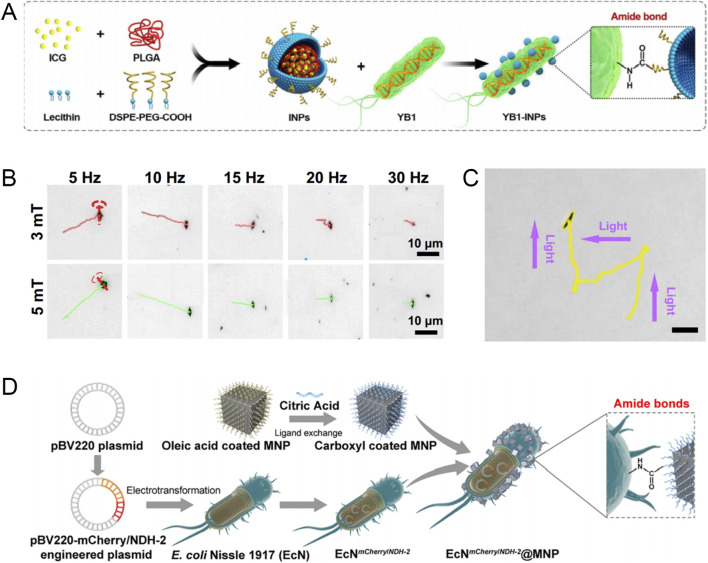
**(A)** Schematic diagram of the preparation process of YB1-INPs. Modified and reprinted from ref. (Chen et al., 2019). Reproduced with permission, Copyright 2019, Elsevier. **(B)** The motion trajectory of the hematite microrobot in rotating magnetic fields of 3 mT and 5 mT. Modified and reprinted from ref (Peng et al., 2022). Reproduced with permission, Copyright 2022, Wiley-VCH. **(C)** Light-controlled steering of a single hematite microrobot by negative phototaxis in 1% H_2_O_2_ (scale bars, 10 μm). Modified and reprinted from ref (Peng et al., 2022). Reproduced with permission, Copyright 2022, Wiley-VCH. **(D)** Schematic preparation of engineered bacteriahybrid microrobots. Modified and reprinted from ref. (Chen et al., 2022). Reproduced with permission, Copyright 2022, American Chemical Society.

The swimming trajectory of bacteria can be affected by environmental gradients such as nutrients and oxygen, and they can use their energy to propel themselves. Such bacteria can take advantage of the tumor-specific pathological environmental gradients to accumulate in tumors. Singh et al. reported a biohybrid microswimmer composed of a double emulsion driven by *Escherichia coli* (Singh et al., 2017). The average swimming speed of the microswimmer in the motility medium can reach 6.5 ± 0.8 μm/s, and it can swim toward cancer cells across the microporous membrane barrier along the glucose concentration gradient and deliver tracking fuel to cancer cells, thereby achieving real-time live cell imaging. Subsequently, bacteria attached to the microswimmer will also be internalized and degraded by macrophages. Chen et al. constructed a biological/abiotic cross-linking system (YB1-INPs) for the treatment of solid tumors, in which the essential gene asd of *Salmonella typhimurium* YB1 (YB1) was replaced by a gene structure controlled by a hypoxia-targeting promoter, which endowed YB1 with excellent hypoxia-targeting and tumor accumulation capabilities. Indocyanine green nanoparticles (INPs) are highly biocompatible photosensitizers. When INPs are covalently linked to the surface of YB1, the hypoxia-targeting ability of YB1 is utilized to allow INPs to effectively accumulate in the hypoxic tumor core. Finally, under the irradiation of the NIR laser, the tumor and the YB1 inside the tumor are simultaneously eliminated (Figure 3A) (Chen et al., 2019).

Sperm is a highly specialized and self-propelled cell that is particularly well suited to move in the female reproductive tract, so transforming sperm into MNRs is well suited to perform functions in the female reproductive tract. Xu et al. successfully loaded doxorubicin (DOX) into human sperm and constructed a sperm micromotor (Xu et al., 2020). The activity of sperm can remain unchanged after drug loading. The average movement speed of sperm can still reach 18 ± 5 μm/s after 1 h of DOX loading, which is the same as before loading the drug. At the same time, the sperm micromotor showed a strong killing effect on HeLa cells and 3D ovarian cancer cells.

Although bioenergy-driven MNRs have shown great advantages in biocompatibility and unique targeting, some issues still need to be considered before clinical application. Although microorganisms can be killed and degraded by the body’s immune system, chemotherapy drugs still pose certain safety risks to cancer patients who are receiving chemotherapy drugs because they kill normal immune cells while fighting tumors. If bacterial MNRs are only used in non-sterile body cavities, the risk of infection may be reduced.

### 2.4 Hybrid-driven micro-/nanorobot

To achieve stronger thrust and more complex medical functions, many researchers use hybrid power to drive micro-/nanorobots. Among them, the use of magnetic field drive combined with other driving methods to propel micro-/nanorobots is the most common method in the diagnosis and treatment of malignant tumors.

The driving method of magnetic field combined with other physical external fields has the characteristics of non-invasiveness, remote controllability, and sensitive operation. For example, in the work of Peng et al., a hematite microrobot with a dendritic structure in a magnetic/optical dual propulsion mode was proposed (Peng et al., 2022). The robot exhibits negative phototropism under light and can be affected by an external rotating magnetic field to perform controllable movement along a predetermined path. The magnetic/optical dual propulsion mode can generate a powerful fluid flow to propel the robot. At the same time, *in vitro* experiments have shown that the robot can produce abundant reactive oxygen species based on the Fenton reaction, which enhances the efficacy of photodynamic therapy on prostate cancer cells (Figures 3B, C). Tang et al. proposed a CAR T microrobot (M-CAR Ts) with artificially modified immunomagnetic beads, which can maintain a predetermined route under magnetic guidance, show excellent acoustic controllability, and actively penetrate tumor tissue under magnetic/acoustic sequential drive. Further experiments showed that anti-CD3/CD28 immunomagnetic beads can significantly enhance the anti-cancer efficacy of CAR T cells (Tang et al., 2023).

The engineered bacterial hybrid microrobot based on magnetic nanomaterials has two propulsion control systems: magnetic guidance and bacterial tropism, which improves the propulsion speed and response speed. For example, Chen et al. conjugated magnetic nanoparticles (MNP) to non-pathogenic bacteria EcN through amide bonds to obtain bacterial hybrid microrobots (EcN@MNP) (Chen et al., 2022). EcN@MNP achieves positive migration ability against tumors through magnetotaxis and hypoxia sensing (Figure 3D).

Although hybrid microrobots constructed using engineered bacteria have excellent propulsion effects and tumor-killing capabilities, there are still some limitations to the application of bacteria in living organisms. Therefore, researchers have turned their attention to macrophages in the collective immune system. Macrophages have an innate phagocytic function and can be used to make eukaryotic cell-based microrobots by endocytosing nanoparticles (Song et al., 2022). Macrophages are circulating cells that can penetrate blood vessels and invade tumors. The researchers loaded citric acid-coated MNPs into primary mouse macrophages to construct a macrophage-based dual-targeted microrobot, which has both the inherent tumor-homing ability of macrophages and can be manipulated by external magnetic fields. Tumor targeting experiments have shown that the microrobot can penetrate the tumor spheres, and this effect is enhanced with the addition of magnetic fields (Nguyen VD. et al., 2021).

## 3 Micro-/nanorobot for cancer diagnosis and treatment

### 3.1 Imaging and tumor diagnosis

Early diagnosis of cancer can greatly improve the patient’s prognosis (Loomans-Kropp et al., 2022), but many malignant tumors have hidden early symptoms and lack effective detection methods, causing patients to miss the best time for treatment. Although tumor markers and imaging diagnosis can detect some early tumors, MNR improves the sensitivity and specificity of early cancer diagnosis due to its small size, flexible controllability, and ability to perform multiple medical functions (Maheswari et al., 2018; Peng et al., 2018).

**FIGURE 4:**
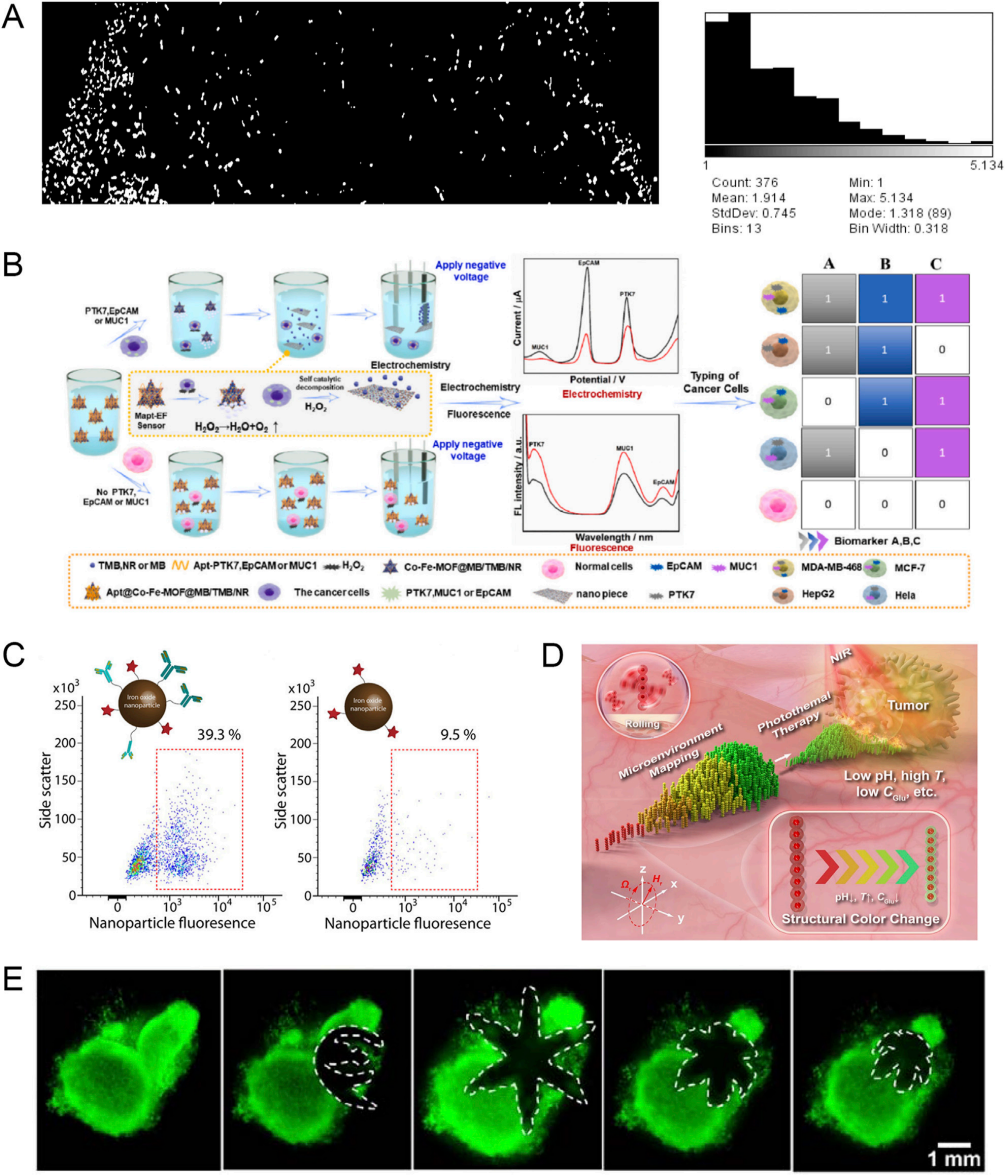
**(A)** The living robot responds to the VEGF gradient in a homemade microfluidic chip. The left channel of the chip is filled with VEGF solution (10µg/10 µL) and the right channel is filled with buffer solution (deionized water). Modified and reprinted from ref (Al-Fandi et al., 2017). Reproduced with permission, Copyright 2017, by the authors. Licensee MDPI, Basel, Switzerland. **(B)** Schematic diagram of using microswimmer cell sensors to detect multiple biomarkers and then classify cancer cells. Modified and reprinted from ref (Zhang Y. et al., 2023). Reproduced with permission, Copyright 2023, Elsevier. **(C)** Microswimmers release anti-ErbB2-modified magnetic nanoparticles to target and label SKBR3 cells. Modified and reprinted from ref (Ceylan et al., 2019). Reproduced with permission, Copyright 2019, American Chemical Society. **(D)** Schematic diagram of RPNRs visualizing tumor lesions based on changes in local physicochemical properties. Modified and reprinted from ref (Li L. et al., 2023). Reproduced with permission, Copyright 2023, The Author(s). **(E)** The gripper captures and excises cells from a live cell mass. Modified and reprinted from ref (Breger et al., 2015). Reproduced with permission, Copyright 2015, American Chemical Society.

During the occurrence and development of malignant tumors, a class of substances produced by the tumor itself that can reflect the existence and growth of the tumor can be considered tumor markers. MNR can intercept this signal change in the body and achieve early diagnosis of cancer. For example, researchers have designed a new type of intelligent DNA nanorobot, using molecular programming and logic gate operations based on toehold-mediated strand displacement reaction to simultaneously detect two tumor miRNAs, namely, miR-21 and miR-125b. In addition, DNA nanorobots were used to seal the pores of DOX-loaded silica nanoparticles. When the target miRNA is present, the drug will be released, which can achieve efficient detection of two tumor miRNAs and tumor killing (Mirzaiebadizi et al., 2022). When tumor cell clusters progress to solid tumors, tumor cells produce a large amount of vascular endothelial growth factor (VEGF) to promote angiogenesis. To detect VEGF early, researchers designed non-pathogenic *E. coli* equipped with a naturally synthesized bio-nanosensor system. The living robot has a chemotactic effect on VEGF, which can be used to detect early cancer (Figure 4A) (Al-Fandi et al., 2017).

Circulating tumor cells (CTCs) refer to tumor cells that fall off from the primary lesion and enter the blood circulation during tumor formation or progression. The detection of CTCs can assist clinicians in diagnosing tumors. However, since CTCs are relatively rare, with an average of only 1–10 CTCs per milliliter of blood, and the presence of normal blood cells can also interfere with current detection methods, there is an urgent need to study detection methods that improve CTC enrichment efficiency and detection sensitivity. Zhang et al. proposed a novel microswimmer dual-mode aptamer (electrochemical and fluorescent) homogeneous cell sensor that can be used to simultaneously detect three biomarkers: PTK7, EpCAM, and MUC1(87). After the markers are detected, the electrochemical and fluorescent signal intensities increase and the sensor can recognize two output signals to identify five different malignant tumor cells. This method has the potential to efficiently identify CTCs (Figure 4B). The glucose level in cancer cells is usually higher than that in normal cells. Dolev et al. took advantage of this feature to design a nanorobot that can examine CTCs by increasing the glucose-driven current in cancer cells and can expose drugs to the tumor site under the action of the driving force (Dolev et al., 2019).

In addition to detecting tumor markers and CTCs to indirectly detect cancer, MNR can also detect primary tumors to directly detect cancer. Taking advantage of the high expression of matrix metalloproteinase-2 (MMP2) in local tumors, the researchers designed a hydrogel microrobot based on MMP2 environmental sensing to achieve diagnostic functions (Ceylan et al., 2019). When the microrobot reaches the periphery of the tumor under the control of an external magnetic field, the local pathological concentration of MMP2 can cause the hydrogel microrobot to expand rapidly and increase the mesh size, and then the magnetic contrast agent labeled with anti-ErbB2 antibodies is released into the local environment to target and label SKBR3 cancer cells with high ErbB2 expression (Figure 4C).

Due to the special metabolism and proliferation patterns of tumor cells, the physical and chemical properties in the tumor microenvironment are somewhat different from those of normal tissues. Exploring these differences in physical and chemical properties can provide clues for tumor diagnosis, understanding pathological processes, studying pathogenesis, and developing effective drugs. Li et al. developed magnetically propelled responsive photonic nanorobots (RPNRs) that can perform controllable collective motion in complex environments and then visualize tumor lesions by mapping local abnormal physicochemical changes (such as pH, temperature, or glucose concentration changes) through their responsive structural colors (Li L. et al., 2023). The structural colors of RPNRs are in the visible light range, so they can be directly used in organs that can be endoscopically viewed, such as the digestive tract, respiratory tract, and bladder (Figure 4D).

### 3.2 Precision surgery

Different from traditional surgery, micro-/nanorobot limits the size of surgery to the cellular level, which is an area that traditional surgery cannot reach (Sun et al., 2020). This makes surgery using MNRs precise, less invasive, and quick to recover.

Currently, in the field of tumor surgery, the main application scenarios of MNRs are single-cell biopsy (Jin et al., 2020) and drilling (Xi et al., 2013). Many tumor lesions are highly heterogeneous at the single-cell level, and operations at the single-cell level can reduce invasiveness and improve accuracy. Breger et al. designed a magnetic field-driven thermally responsive gripper (Breger et al., 2015). The gripper is fully folded in one direction in a cold storage fluid and then deployed to the desired part of the body using a catheter. When the gripper transitions from a cold state to a physiological temperature, a self-folding transition occurs, through which cells in fibroblast bundles can be grasped and removed. The gripper is finally retrieved using an external magnetic field and catheter (Figure 4E). In addition, other research teams have also achieved the extraction of intact cells from free pig liver tissue (Gultepe et al., 2013) and esophagus (Ghosh et al., 2021), showing great potential for clinical application. In addition to single-cell biopsy, MNR can also drill holes in single cells and open cell membranes to create cell incisions. Researchers have designed a “dual-function micro-dagger” that provides the dual functions of cell membrane drilling and drug release to achieve non-invasive surgery and precise killing of single cells (Srivastava et al., 2016).

Although single-cell level operations cannot be extended to clinical practice today, precision tumor surgery offers distinct advantages that cannot be achieved through traditional surgical techniques. We believe that this is a research direction with potential and development.

### 3.3 Drug delivery

Drug delivery and targeted therapy are the core functions of using MNRs to treat malignant tumors. Under traditional drug delivery methods, the amount of effective drug reaching the target area is less than 1% of the total amount of drug administered. To achieve the target treatment effect, side effects will increase significantly. Using MNRs to deliver drugs can target specific lesions to achieve directional and controllable drug release, which can effectively avoid the high doses and serious adverse reactions of traditional tumor treatments and achieve targeted drug delivery.

**FIGURE 5:**
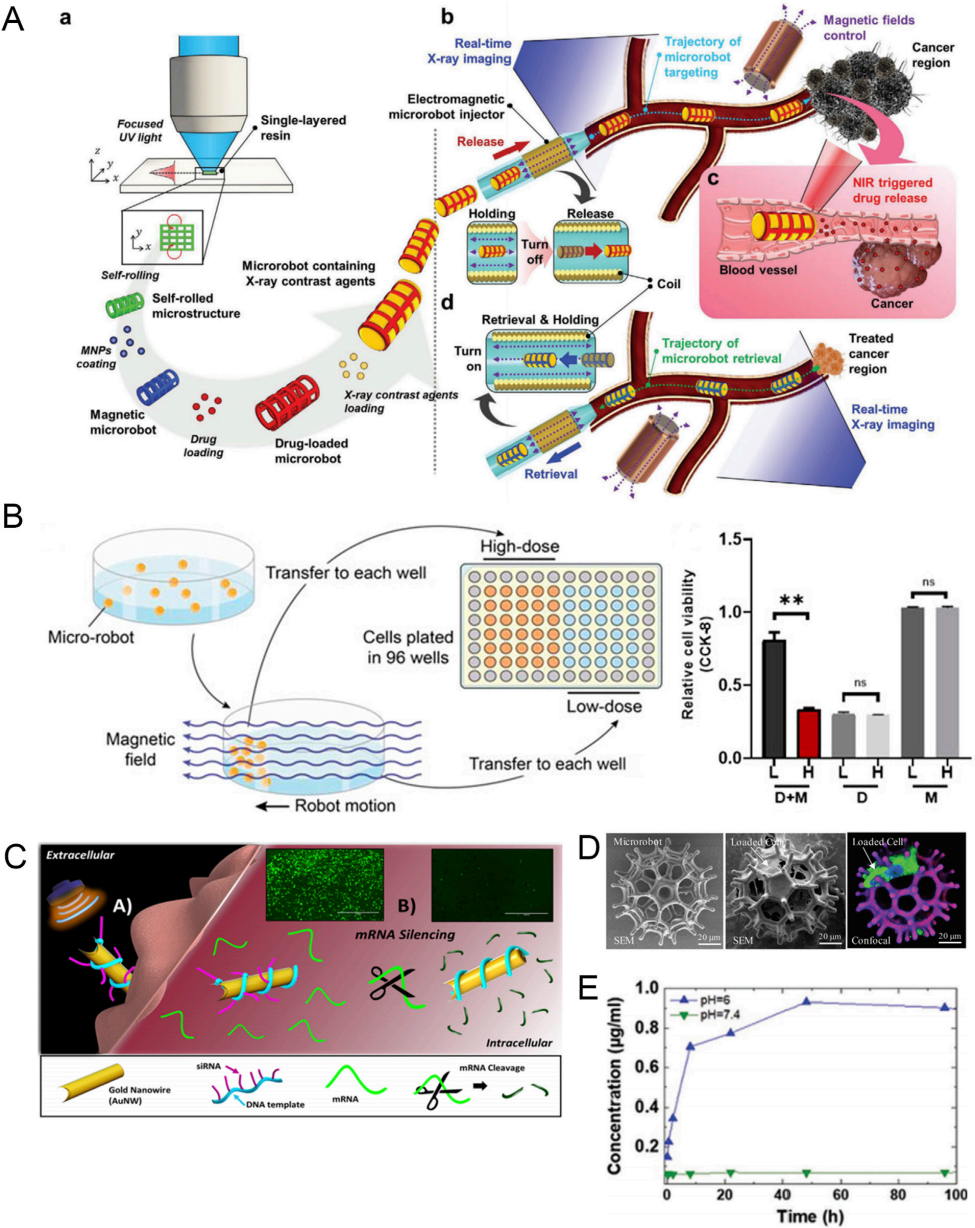
**(A)** Schematic diagram of the assembly, imaging, drug release, and automated retrieval of the targeted drug delivery microrobot. Modified and reprinted from ref (Nguyen KT. et al., 2021). Reproduced with permission, Copyright 2021, Wiley-VCH. **(B)** *In vitro* experiments of drug-loaded hydrogel microrobots. D + M, Microrobots with EPZ015666; D, Dissociative EPZ015666; M, Drug-free microrobots. Modified and reprinted from ref (Mu et al., 2022). Reproduced with permission, Copyright 2022, The Authors. **(C)** Schematic diagram and fluorescence images of nanomotor silencing genes (scale bars, 1,000 μm). Modified and reprinted from ref (Esteban-Fernández de Ávila et al., 2016). Reproduced with permission, Copyright 2016, American Chemical Society. **(D)** SME and confocal images of the microrobot when empty and loaded with cells. Modified and reprinted from ref (Wei et al., 2020). Reproduced with permission, Copyright 2020, Wiley-VCH. **(E)** The release amount of DOX in buffer solutions with different pH values. Modified and reprinted from ref (Terzopoulou et al., 2020). Reproduced with permission, Copyright 2020, Wiley-VCH.

When using MNRs to treat malignant tumors, the most common cargo loaded on MNRs is chemotherapy drugs. For example, Nguyen et al. developed a magnetically guided microrobot consisting of a ceramic-based self-rolled body and an MNP coating (Nguyen KT. et al., 2021). Because of the porous shape and large surface area of the microrobot body, it can support high-load MNP, drugs (DOX), and X-ray contrast agents, making it have functions such as magnetic control, killing, and real-time imaging under X-ray. The researchers verified the effect of blood flow on the performance of the microrobot in the body by constructing a fluid channel. The results showed that the microrobot can smoothly use an external magnetic field to control its precise reach to the lesion site and release drugs under real-time X-ray imaging (Figure 5A). Park et al. designed a magnetic nanoparticle encapsulated with DOX and magnetite, which combined with *E. coli* and used magnetic guidance and chemotaxis to precisely target 4T1 tumor cells. Compared with the conventional drug treatment group, this smart drug delivery system increased the accumulation of DOX in 4T1 cells and enhanced the anti-cancer efficiency (Park et al., 2017).

In addition to common chemotherapy drugs, MNRs can also be loaded with inhibitors or siRNA targeting specific targets. Although such inhibitors and siRNA can significantly inhibit specific targets in tumor cells and kill tumors, normal cells will also be affected and cause strong side effects. Therefore, researchers have designed a precise delivery system based on MNRs to reduce side effects and improve treatment efficiency. Mu et al. developed a novel hydrogel microrobot to deliver a PRMT5 inhibitor EPZ01566, which can selectively inhibit the growth of MTAP-deficient osteosarcoma under the control of an external magnetic field (Figure 5B) (Mu et al., 2022). To perform RNA interference at the post-transcriptional level and inhibit the expression of specific proteins, researchers designed a self-propelled nanowire using the siRNA-DNA nanotechnology platform to deliver siRNA inside cells. The study used green fluorescent protein-targeted siRNA (siGFP), which can cut the target mRNA when entering the cell, thereby silencing the formation of new fluorescent proteins. The rapid disappearance of green fluorescence in the experiment can reflect the effective delivery of siRNA inside the cell. Driven by ultrasound, the acoustic nanomotor can silence HEK293-GFP cells up to 94% (Figure 5C) (Esteban-Fernández de Ávila et al., 2016). This provides an outstanding smart delivery platform for the precise delivery of siRNA to tumors.

In addition to being loaded with chemotherapy drugs and therapeutic compounds, micro-/nanorobots loaded with therapeutic stem cells can solve the problem of current cell therapies being unable to target therapeutic cells to diseased parts of the body (Jeon et al., 2019). For example, Wei et al. developed a magnetically driven microrobot with a burr-like porous spherical structure and carried human induced pluripotent stem cell-derived mesenchymal stem cells that can deliver glutathione peroxidase 3 (hiPSC-MSC-GPX3), which plays a role in inhibiting cancer cell proliferation in cancer treatment (Wei et al., 2020). Under the action of an external magnetic field, the microrobot can achieve automatic navigation for cell delivery in vascular tissue. In vivo experiments, the engineered stem cells released by the microrobot can significantly reduce the growth of orthotopic liver tumors (Figure 5D).

To achieve targeted tumor therapy using MNR carriers, appropriate drug release is as important as efficient delivery. Reasonable drug release can not only increase local drug concentration but also reduce damage to normal cells. The most common mode of local drug release in tumors is pH-responsive release (Xin et al., 2021; Darmawan et al., 2022). For example, researchers designed a small biomedical robot based on a metal organic framework (MOFBOTs) and studied the drug release pattern of the robot’s drug delivery component (Fe@ZIF-8) by comparing the drug release of Fe@ZIF-8-loaded DOX under acidic (pH = 6, which is also the extracellular environment of tumor cells) and physiological (pH = 7.4) conditions. At acidic pH, DOX was rapidly released within the first 12 h, and complete drug release was observed within 48 h. In contrast, no DOX release was observed within 96 h at physiological pH. This shows that Fe@ZIF-8 can maintain stable drug loading under physiological conditions and release drugs under acidic conditions in the tumor matrix (Figure 5E) (Terzopoulou et al., 2020). In addition to pH-responsive release, there are also drug release modes that are triggered by temperature (Zhou et al., 2023) and light (Sridhar et al., 2022). Therefore, not only can the physicochemical properties of the tumor microenvironment be used to design drug-responsive release, but changes in external conditions can also be artificially created to promote drug release.

The drug delivery targeted therapy mode provided by MNR is expected to change the current situation of low drug delivery efficiency of traditional drug delivery modes, realize the efficient transportation and conditional release of drugs or other therapeutic substances, and provide a solution for “real” targeted therapy in tumor treatment.

## 4 Conclusion and perspectives

MNR is one of the most promising tools in nanomedicine (Zhang et al., 2025; Yu S. et al., 2022). It can optimize the current early diagnosis and precision treatment strategies in the field of tumors, thereby improving the prognosis of tumor patients. In recent years, with the development of nanotechnology and other multidisciplinary disciplines, the use of MNRs in tumor treatment has evolved from theory to practice and has gradually matured. However, further development and verification are still needed from the laboratory to the actual clinical treatment of patients. This review starts with the driving mode of MNR and its application in the field of tumors and reviews the development process and latest progress of MNR in recent years.

Although MNRs have made encouraging progress in treating malignant tumors *in vitro* and *in vivo*, there are still some limitations in their applications:

(1) Although the driving of micro-/nanorobots has achieved remote control and movement in low Reynolds number environments, solutions are still needed to solve the problems of byproducts generated to generate propulsion, the ability of external fields to penetrate tissues, and the damage of high-intensity external fields to normal tissues. Therefore, we should not be limited to the current driving methods of micro-/nanorobots but should explore new driving modes and driving combinations.
(2) Although *in vitro* and *in vivo* experiments have confirmed that MNR can accurately reach the tumor site under the control of driving force, different tumors are located in different organs and have different physiological and pathological characteristics and environments. Therefore, it is necessary to optimize the targeting path according to the part of the body where the tumor is located, and even design individualized solutions according to the different conditions of different patients. At the same time, we should also explore an MNR real-time tracking system that is more suitable for clinical use. The establishment of such a visualization system can improve treatment accuracy and shorten the average treatment time of patients.
(3) At present, most strategies for treating tumors with MNRs are single-drug delivery solutions. However, due to the heterogeneity and drug resistance of tumors, tumor cells may not be sensitive to the delivered drugs, resulting in insufficient treatment intensity. Therefore, in the process of delivering drugs to treat tumors, the driving mode can be combined to enhance the tumor treatment effect. For example, light-field-driven MNRs can be combined with photothermal or photodynamic therapy; chemical-driven MNRs can be combined with chemodynamic therapy to enhance the tumor-killing effect.
(4) When using non-organic materials to treat diseases, the inevitable topic is biosafety, especially for MNRs. In addition, MNRs also involve mechanical failure issues. Therefore, when designing and testing MNRs, materials with good biocompatibility are selected, and strict quality control is performed on MNRs to reduce their failure rate to an acceptable range. In addition, due to their excellent biocompatibility, even if a failure occurs, it will not affect the normal function of the body.

Although many technical problems still need to be solved before MNRs can be applied to clinical diagnosis and treatment, we believe that with the multidisciplinary cooperation and development related to MNRs, researchers can overcome the difficulties and enable MNRs to perform more and more complex medical tasks, providing more precise treatment for future cancer patients.

## Abbreviations

AIE, aggregation-induced emission; CTCs, circulating tumor cells; DOX, doxorubicin; MNR, micro-/nanorobot; UV, ultraviolet; PHI, polyheptazine imide; NIR, near infrared; NR, nanorocket; RMF, rotating magnetic fields; MTB, magnetotactic bacteria; *salmonella* YB1, typhimurium YB1; INPs, indocyanine green nanoparticles; MNP, magnetic nanoparticles; VEGF, vascular endothelial growth factor; MMP2, matrix metalloproteinase-2.
